# NGMD: next generation malware detection in federated server with deep neural network model for autonomous networks

**DOI:** 10.1038/s41598-024-61298-7

**Published:** 2024-05-13

**Authors:** Himanshi Babbar, Shalli Rani, Wadii Boulila

**Affiliations:** 1 Chitkara University Institute of Engineering and Technology, Chitkara University, Punjab, India; 2 Robotics and Internet-of-Things Laboratory, Prince Sultan University, 12435 Riyadh, Saudi Arabia; 3 RIADI Laboratory, National School of Computer Sciences, University of Manouba, 2010 Manouba, Tunisia

**Keywords:** Electrical and electronic engineering, Energy science and technology

## Abstract

Distributed denial-of-service (DDoS) attacks persistently proliferate, impacting individuals and Internet Service Providers (ISPs). Deep learning (DL) models are paving the way to address these challenges and the dynamic nature of potential threats. Traditional detection systems, relying on signature-based techniques, are susceptible to next-generation malware. Integrating DL approaches in cloud-edge/federated servers enhances the resilience of these systems. In the Internet of Things (IoT) and autonomous networks, DL, particularly federated learning, has gained prominence for attack detection. Unlike conventional models (centralized and localized DL), federated learning does not require access to users’ private data for attack detection. This approach is gaining much interest in academia and industry due to its deployment on local and global cloud-edge models. Recent advancements in DL enable training a quality cloud-edge model across various users (collaborators) without exchanging personal information. Federated learning, emphasizing privacy preservation at the cloud-edge terminal, holds significant potential for facilitating privacy-aware learning among collaborators. This paper addresses: (1) The deployment of an optimized deep neural network for network traffic classification. (2) The coordination of federated server model parameters with training across devices in IoT domains. A federated flowchart is proposed for training and aggregating local model updates. (3) The generation of a global model at the cloud-edge terminal after multiple rounds between domains and servers. (4) Experimental validation on the BoT-IoT dataset demonstrates that the federated learning model can reliably detect attacks with efficient classification, privacy, and confidentiality. Additionally, it requires minimal memory space for storing training data, resulting in minimal network delay. Consequently, the proposed framework outperforms both centralized and localized DL models, achieving superior performance.

## Introduction

The most destructive cyberattacks, particularly DDoS assaults, continue to inflict unintended damage on ISPs and network operators, ranking among the top security concerns^1^. Recent catastrophic events, characterized by traffic attacks exceeding 1 Tbit/s, underscore the increasing strength, sophistication, and devastating impact of DDoS attacks. Annually, these substantial security risks impose significant financial losses on both commercial and academic institutions. Large-scale and severe DDoS attacks are exacerbated by the proliferation of IoT botnets and the rapid growth in unsecured IoT devices, projected to reach 10 billion by 2027^2^. This escalating trend poses a considerable threat, contributing to the intensification of DDoS attacks. The need for robust defense mechanisms is evident, and the significance of advancements in detection and prevention methods, such as the proposed federated learning model, becomes increasingly apparent in the face of evolving and more potent cyber threats. The development of new cyberattack techniques, such as botnet as a service, gives attackers more sophisticated tools with which to launch new attacks that may result in various types of damage, such as the theft of sensitive data, the disruption of some crucial industries (such as healthcare and finance), and network intrusion. As a result, there is a critical need for extremely powerful detection systems^3^ to protect networks against DDoS attacks and other types of intrusive activity. Detection systems are important for maintaining information security and are regarded as the first line of defense against network security risks; they work to quickly and accurately identify such threats while producing a few false positives. Conventional detection systems, on the other hand, are pattern- and signature-based detection systems, which makes it challenging to find autonomous assaults.

Furthermore, effort is needed to constantly enhance and evolve such detection systems to detect next-generation malware.

Fifth-generation (5G)^4^ cellular networks are currently advancing and putting different demands on them, notably those for dependability, delay, and throughput. Confidentiality is another issue that has drawn the most interest from the networking and telecommunications research community as networks are becoming more complicated. The concept of mobile networks that go beyond 5G or 6G considers crucial characteristics like intelligent security and automated security management. Implementing such security monitoring solutions and their maintenance with minimal to no human interaction while meeting high-performance standards would provide a plethora of novel obstacles. To satisfy the automation needs of network administration, the European Telecommunications Standards Institute (ETSI) established the Zero-touch Network (AN) architecture. The AN design, which divides the network into clusters depending on their various requirements, such as logistical, commercial, and technical demands, also includes closed-loop operations and artificial intelligence (AI) and machine learning (ML) techniques. The basic AN architecture^5^, safety closed-loop activities for detecting attacks, security assessment, cyber threat intelligence for threat mitigating or avoidance, safety coordination, and defense policy modifications can all be directly impacted by safety constraints.

The authors in^6^, construct smart transactions and keep hostile or unreliable participants out of FL, suggest a blockchain- based safe FL architecture. In order to prevent poisoning attempts, the central aggregator distinguishes hostile and unreliable individuals by automatically executing smart contracts. Furthermore, we defend against membership inference attacks using local differential privacy approaches. In^7^, every UAV has a Computing Element (CE) that handles tasks that are sent to it via vertical offloading from the equipment on the ground. In order to ensure that the FANET processing latency for each work acquired is reduced and is almost independent of the activity status of the area encompassed by the UAV collecting that job, horizontally offload amongst UAVs of the FANET is also added for load balancing considerations. Deep Reinforcement Learning (DRL) is the foundation of the suggested FANET management architecture, enabling zero-touch adaptation to the time-variant activity state of the region serviced from each UAV. As seen by the various researchers^8^, there should be a sufficient number of detectors powered by ML techniques in the network inspired by the AN design to evolve in various fields, including image and speech recognition. Detectors will nevertheless have limitations in terms of processing speed and storage. In this detection, systems have acquired these techniques to identify the security attacks of the traffic on the network. Without updating the protocols of conventional IDS, the IDS is able to detect existing or any new attacks^9^. These ML/DL-based detection systems are ineffective when dealing with newly emerging security threats since there aren’t enough balanced and recently labeled training datasets. To overcome all these issues, the concept of federated learning (FL)^10^, a comparatively recent type of machine learning algorithm, permits decentralized processing with greater privacy and communication effectiveness. In FL, the models are aggregated after being trained on decentralized devices^11^. In FL, every contributor trains locally a globally interconnected model utilizing its local training data, then provides just the local model updates, as opposed to transporting sensitive information of every collaborator to a centralized authority (e.g., server) in order to build a specific ML model. Even though only short model updates are exchanged, as opposed to transmitting the complete raw training data across the network, FL can dramatically reduce privacy issues and communication costs (e.g., network bandwidth utilization)^12^.

## Motivation

Federated learning has demonstrated its capability to enhance vast, unstructured, and diversified datasets in zero-touch networks. This is particularly advantageous as it automatically facilitates learning at both local (local server) and global (cloud edge terminal) levels, enabling the extraction of hidden patterns from massive data volumes. To ensure network security, as highlighted by^13^, the concept of a detection system can leverage this approach to handle unforeseen malicious data effectively. The deployment of an intrusion detection system becomes imperative for identifying and distinguishing between normal and malicious traffic on the network^14–16^. Compared to other learning techniques, a federated learning-based detection system significantly improves overall accuracy^17–19^.

While much of the existing research in 4G and 5G communications focuses on network performance, dependability, and delay, attention to network security and confidentiality in wireless communication has increased in recent years. The safeguarding of data security and privacy has become a crucial aspect of human-centric zero-touch communications, directly impacting users’ lives. Simultaneously, the lawful collection of extensive user information by communication and data service providers often leads to the inadvertent leakage of personal data. Consequently, identifying intrusions with federated learning becomes challenging, particularly in zero-touch networks where applications often involve large sequences of data based on time series.

In conclusion, there is an anticipation that federated learning approaches can be effectively employed on cloud edge terminals to develop intrusion detection-enhanced learning techniques in zero-touch networks, addressing the intricate challenges associated with data security, privacy, and intrusion identification.

## Problem definition

This paper introduces the utilization of Federated Learning (FL) to establish a framework for the anomaly detection of attacks within the architecture of AN. The proposed architecture presents a novel approach incorporating Federated Learning and zero- touch networks, providing an effective security-preserving solution. This framework develops an anomaly detection mechanism specifically designed to manage the DDoS collaboration process among diverse IoT clusters. Firstly, the security and privacy preservation achieved through collaborative learning among various collaborators make it highly effective for the selected collaborative Intrusion Detection System (IDS). Secondly, Federated Learning acts as a deterrent against attacks, as potential attackers may attempt to retrieve crucial information about the training data from locally updated computations transmitted by each collaborator. Thirdly, the evaluation is conducted using the BoT-IoT dataset to secure the network, encompassing parameters such as protocol, source address, source port, destination address, sequence number, standard deviation, source and destination rate, and various attack categories including Denial of Service (DoS), DDoS, Reconnaissance, and Information theft. The evaluation results demonstrate the effectiveness of the proposed architecture compared to state-of-the-art centralized machine or deep learning models concerning accuracy, precision, recall, and F1-score in detecting the number of attacks occurring in different clusters.

## Contributions

The major contributions in the paper are summarized as:

1. Utilizing federated learning, a distributed and secure collaborative framework is proposed to enable diverse IoT domains to construct an effective attack detection model on cloud-edge terminals. This framework is designed to handle authorized attacks, ensuring security while safeguarding the privacy of each domain within the IoT ecosystem.
2. Furthermore, a federated secure server aggregation mechanism is developed to securely aggregate data from each collaborator for updating localized models. A deep neural network architecture is specifically tailored for classifying network traffic, with the FedAvg technique employed for merging updates from local models.
3. To validate the efficacy of the proposed framework, simulations are conducted using the BoT-IoT dataset. Performance evaluation metrics, including accuracy, precision, recall, and F1-score, are utilized across five IoT-based domain devices. The aim is to achieve maximum accuracy, a high rate of attack detection, and privacy preservation to effectively counter the significant scale of security threats in IoT environments.

## Attacks in IoT-based federated learning for zero-touch networks

**Figure 1:**
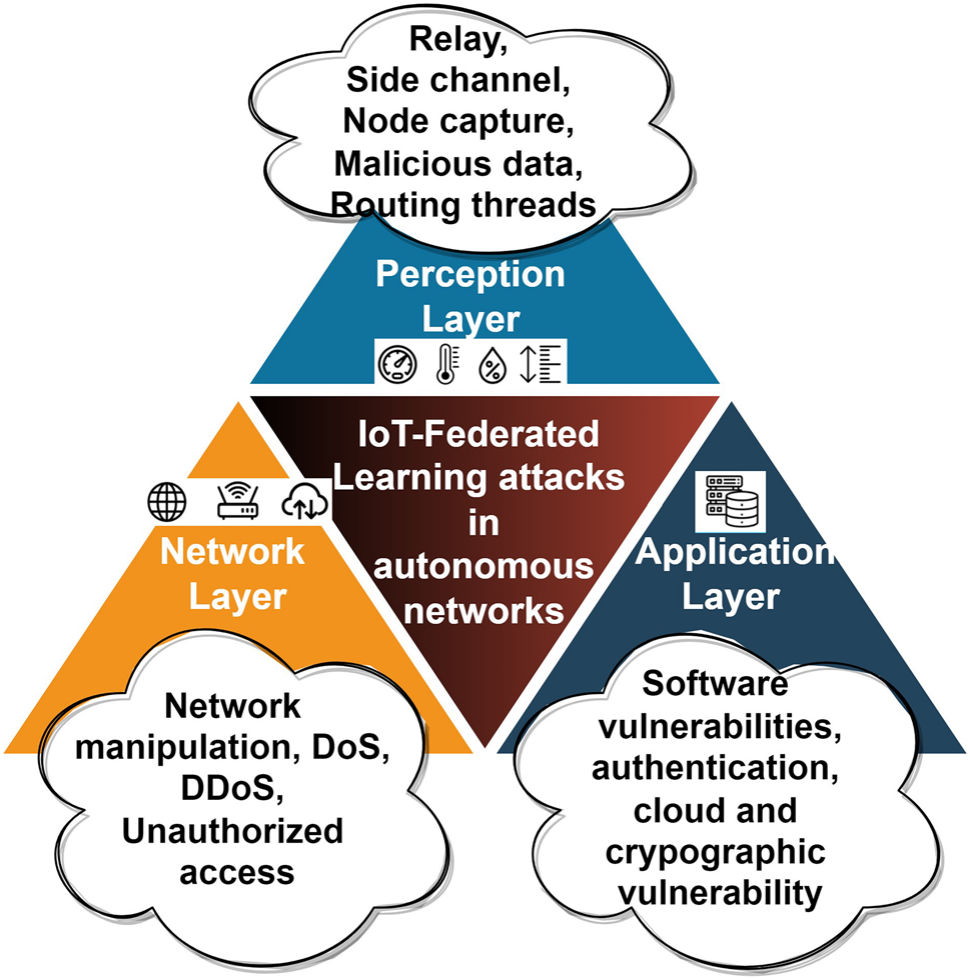
Attacks in IoT-based federated learning.

In this subsection, the main attacks are in multiple layers of IoT-based federated learning for zero-touch networks are discussed (Fig. 1). The three-layered framework has been highlighted to show the attacks on the different layers of IoT-based federated learning zero-touch network system:

In the perception layer, there are numerous resource-restricted sensors and actuators that transmit and retrieve the data with regard to the multiple communication technologies that include Bluetooth, 6LowPAN, etc.^20^. More often all the devices are located at diversified positions, the injection rate is maximum with respect to the captured nodes. This layer is vulnerable to relays, side channels, malicious data, and routing threads. The network layer intimates the information that is directed and sent generously. The information or data is interchanged and may initiate the diversification of vulnerabilities in the network, which includes manipulation in the network, DDoS and DoS attacks, access to authentication and authorization, etc. In the application layer, the top layer, sometimes referred to as “layer 7,” is where business logic is delivered to systems and where user interfaces are provided for traffic control, mobility evaluation, resource endowments, and forecast capabilities. The majority of software weaknesses, such as user acquisition, unprotected login details, and access termination after a predetermined number of failed password guesses, affect the application layer^21^. The various attacks in this layer are authentication, cloud, and cryptographic vulnerabilities and vulnerabilities in the software.

## Proposed methodology

### Federated learning in autonomous networks

**Figure 2:**
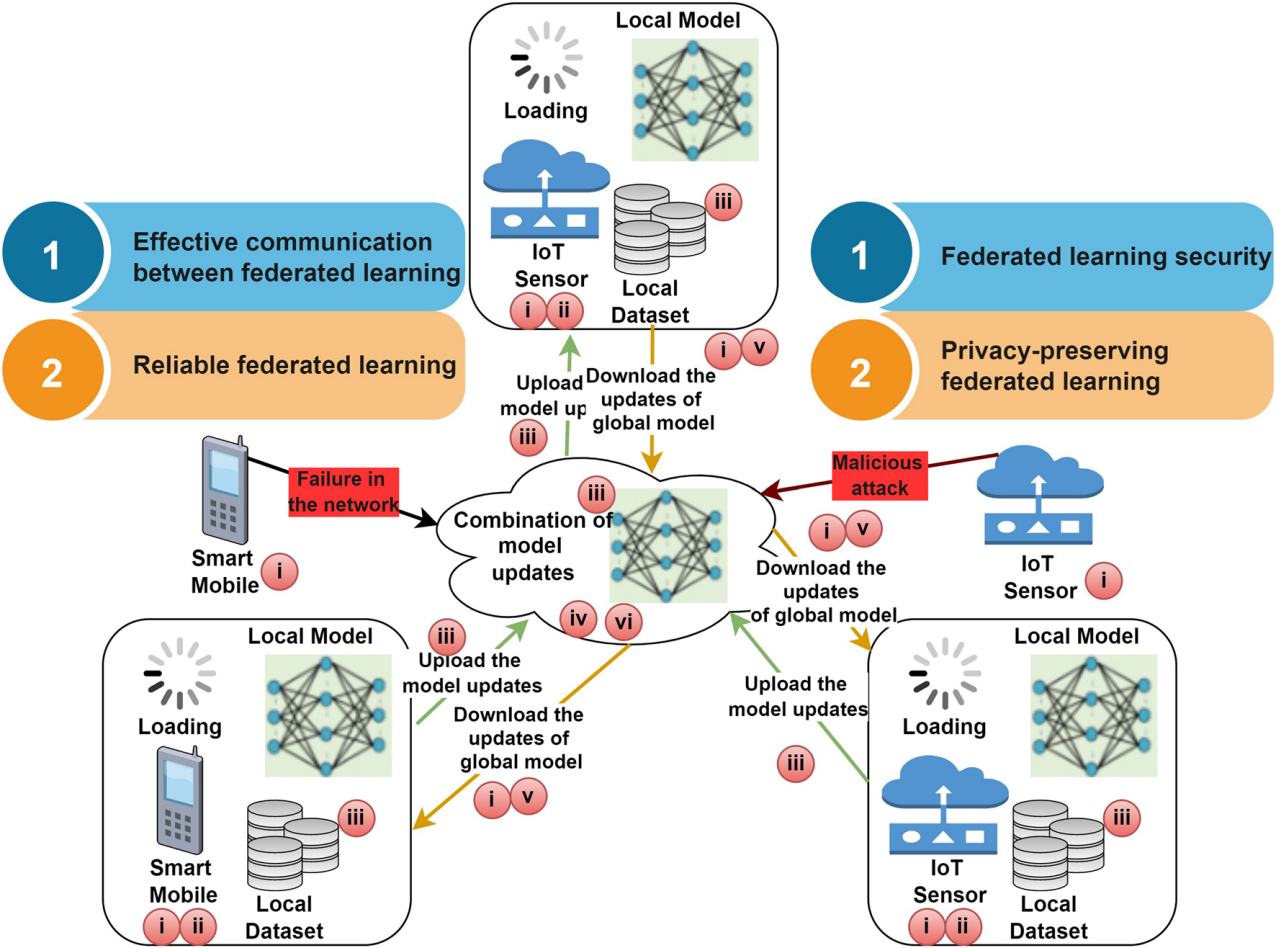
Role of federated learning in autonomous networks.

The framework for federated learning in zero-touch networks comprises various services in which the massive number of decentralized devices can collectively exchange the global model based on anomaly detection by deploying the local datasets. In Fig. 2, the strategy of the federated learning framework is split up into three steps: System Initialization, Training Model, and Aggregation Stage. In the system initialization stage, service requests will be computed by the device and determined to sign up with the accessible cloud to connect the training of the global model through the zero-touch networks. Furthermore, a fraction of the registered devices will be selected randomly by the cloud, serving as task distributors to engage in this round of training. In contrast, the other registered devices will be rejected. The cloud will transmit the initialized or global model that is pre-trained *θ*_*c*_ to every chosen device ((i),(ii)). The next stage, i.e., the training stage, in which each device that is chosen trains the global model *θ*_*m*_← *θ*_*c*_ by deploying the localized dataset for each round, to acquire the updated global model *θ*^*m*^_*c+1*_*.*

In general, *m*th device $\left( {{\text{m}} \in {1},{2},...,M} \right)$ , the optimized loss function is shown as arg min $P_{c} (\theta ),P_{c} (\theta ) = \frac{1}{{E_{c} }}\sum a \in E_{c} p_{a} (\theta )$, in which *E*_*c*_ represents the size of the localized dataset that comprises of input and output pairs of vectors (*k*_*a*_*, j*_*a*_), *k*_*a*_*, j*_*a*_ ∈ *Q, θ* represents the parameter of local model, *p*_*a*_(*θ*) shows the loss function locally. The model updates to the cloud are done in which the updates of each device chosen were uploaded ((iii),(iv),(v)). In the aggregation stage, all chosen devices’ model upgrades are sent to the cloud for consolidation to generate a new global model *θ*_*c*+1_ for the next round in which the number of edge nodes is signified by M. In the next round, the cloud’s chosen device retrieves the most recent global model, *θ*_*c*+1_, from the cloud.

The device will upgrade its particular model using the newly obtained global model. The cloud will choose a random device subset arbitrarily for the subsequent training cycle, and it will continue the previous steps until the trained model satisfies the halting criterion (vi).

### Proposed framework for detection of DDoS attacks in federated learning-based autonomous networks

In this section, the proposed framework ensures secure privacy preservation for collaborative learning within diverse distributed IoT domains. The framework facilitates secure collaboration among various IoT domains. Initially, the company establishes collaboration in the federated server, enabling the learning process to occur collaboratively only with authorized collaborators. Utilizing a federated server ensures the system’s privacy, confidentiality, flexibility, and adaptability. The company initiates global model training across authenticated collaborators. Broadly, the company represents the global model alongside a collection of parameters, transmitting these parameters to each collaborator. The encryption employed aims to prevent any entity, such as an aggregator, from reconstructing a collaborator’s private information from its model notifications. Each collaborator assists in calculating simultaneous local model updates based on its local training data, subsequently transmitting the encrypted values of these updates to secure aggregators. These aggregators amalgamate the local encrypted updates provided by each collaborator, and the combined value is then relayed to the company. Subsequently, the company decrypts the aggregated results and delivers the global model parameters to each collaborator. This iterative process is repeated for a predetermined number of rounds or until a predetermined stop condition is met.

**Figure 3:**
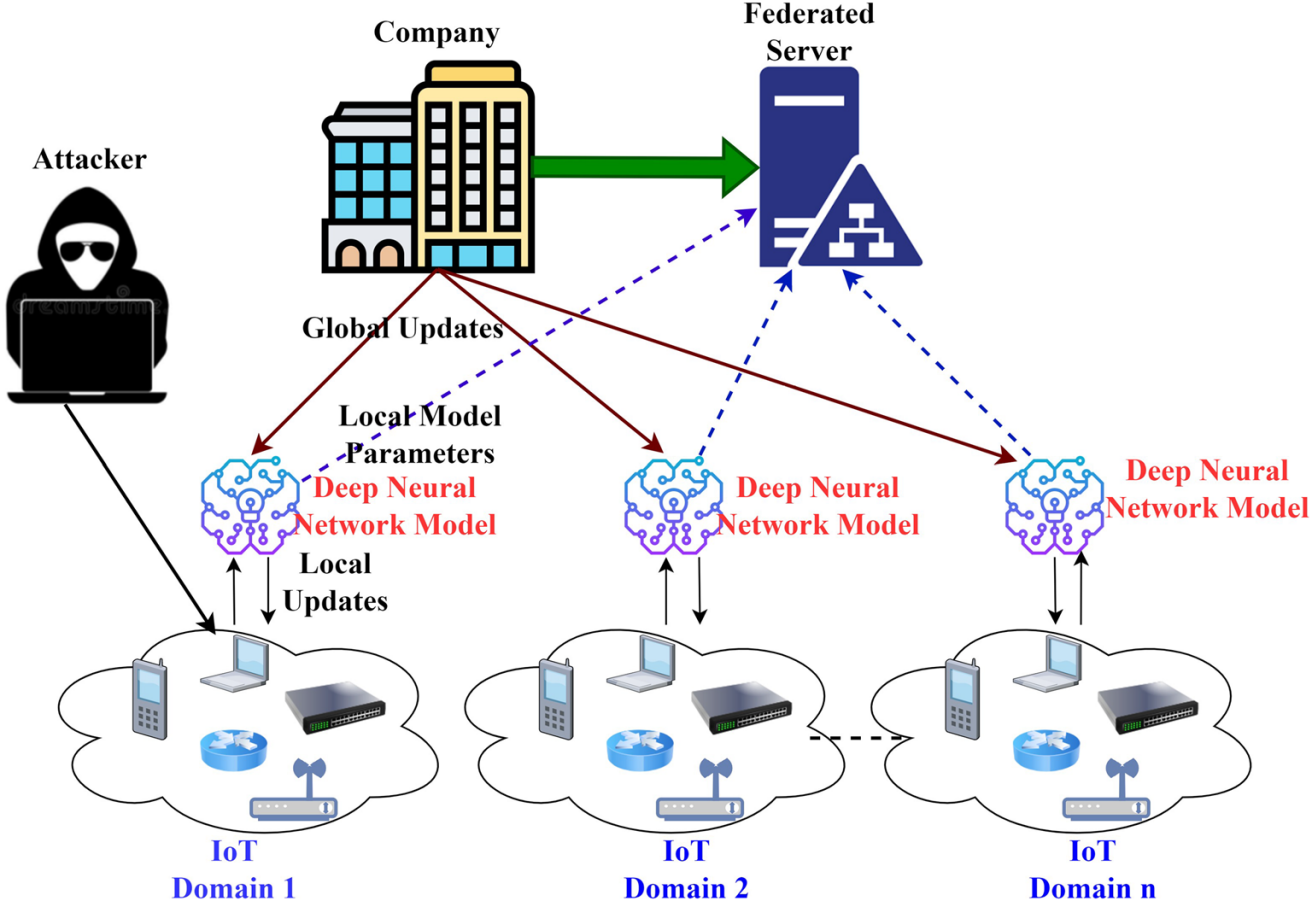
System model of proposed framework.

In the proposed framework, the company manages the secure and private preserving process of collaborative learning amongst various IoT domains^22^. The company generates and utilizes the proposed work in the server. Then, the collaborators in federated learning IoT domains are added to the system of collaboration. The information to be included is the collaborator’s address, which will provide the flexibility for the company to insert or delete the collaborators from the system and manage them in a decentralized, trustworthy way. The framework enables private learning amongst the IoT domains by merging federated learning with the secure federated server. The work is illustrated in Fig 3:

The company, federated secure server aggregator, IoT domains (collaborators). The company starts with collaborative learning by defining the parameters that are at the global model defining the set of weights s0. Later, it chose C-authorized collaborators and delivered the global model to every authorized collaborator. Where every collaborator c, 1 <= *c* <= *C*, executes all the local updates and, based on the global model, evaluates in parallel with its training dataset locally. Every collaborator performs the encryption on the local model updates and divides all these updates among *FS*_*h*_ shares. Every federated secure server aggregator *fs*, 1 <= *f s* <= *FS*_*h*_ acquires any one of the shares of every collaborator and aggregates the acquired encrypted updates of the local model through global model updates, which are later delivered to the company. The company will decrypt the parameters of the global model and initiate the newest round. This procedure is repeated till we reach the maximum round. The controller in domains extracts the features of network traffic for training the local model, once the global model training is completed, the utilization of the global model is to efficiently cope with the security-related threats. The objective function of neural network optimization is:

1 $$ Minimum_{{d_{h} {\kern 1pt} belongs{\kern 1pt} to{\kern 1pt} H^{d} }} f(d_{h} ) = \frac{1}{C}\sum\limits_{c = 1}^{C} {f_{c} } (d_{h} ) $$

The Eq. 1 is explained as C depicting the total number of collaborators for each domain, *f*_*c*_(*d*_*h*_) indicates the objective function performed locally for the *c*^*th*^ collaborator, *f*_*c*_ shows the parameterized function denoted by the high dimensional vector *d*_*h*_ ∈ *H*^*d*^, for every round h, the locally trained comprised of selecting the parameters *d*_*h*_. The minimized local loss function is evaluated as follows:

2 $$ \forall c,f_{c} (dh) = \frac{1}{{n_{c} }}\sum\limits_{{j_{c} }}^{{n_{c} }} { = 1f_{jc} (d_{h} ;a_{jc} ;b_{jc} )} \, $$

where in Eq. 2*n*_*c*_ denotes the local samples (number) with respect to (*a*_*jc*_*, b *_*jc*_) of the *c*th collaborator.

**Figure 4:**
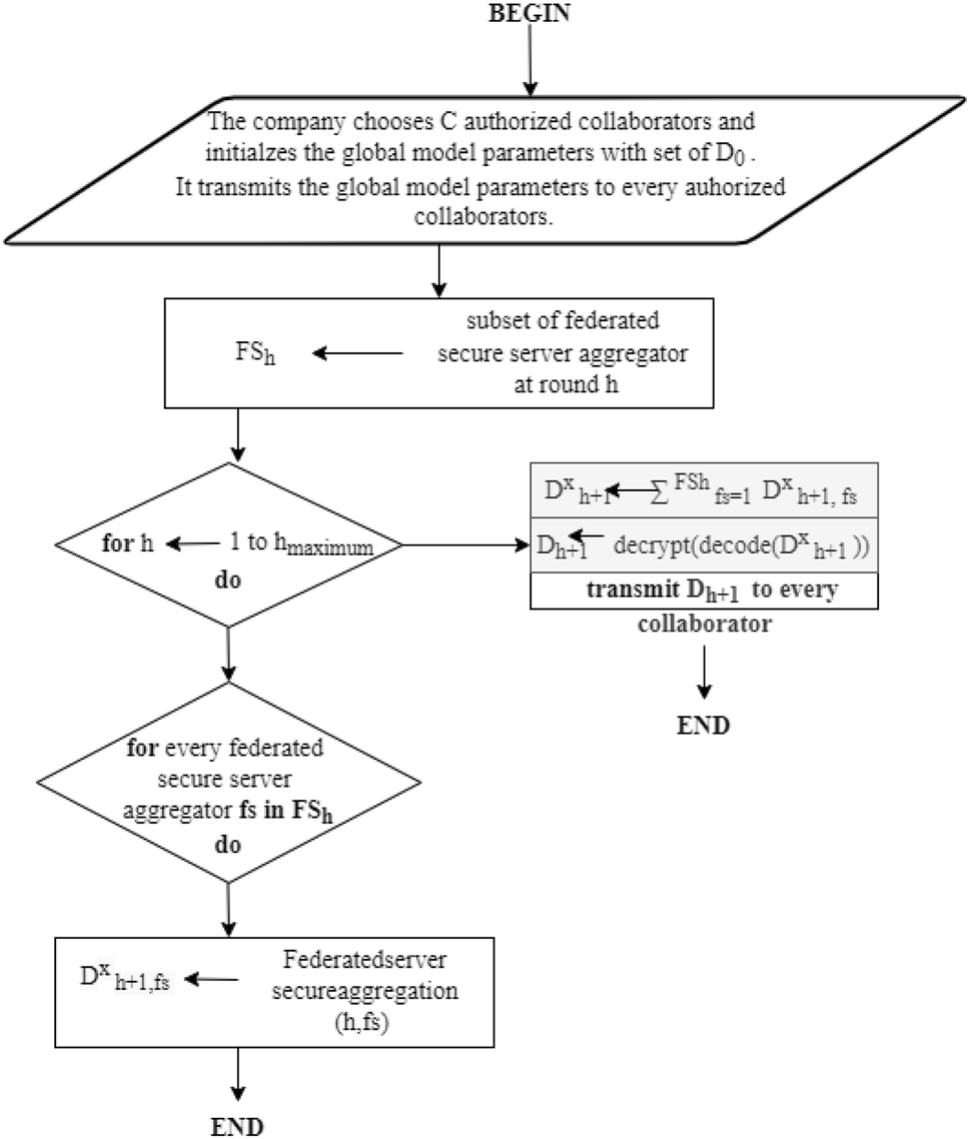
Flowchart for training.

**Figure 5:**
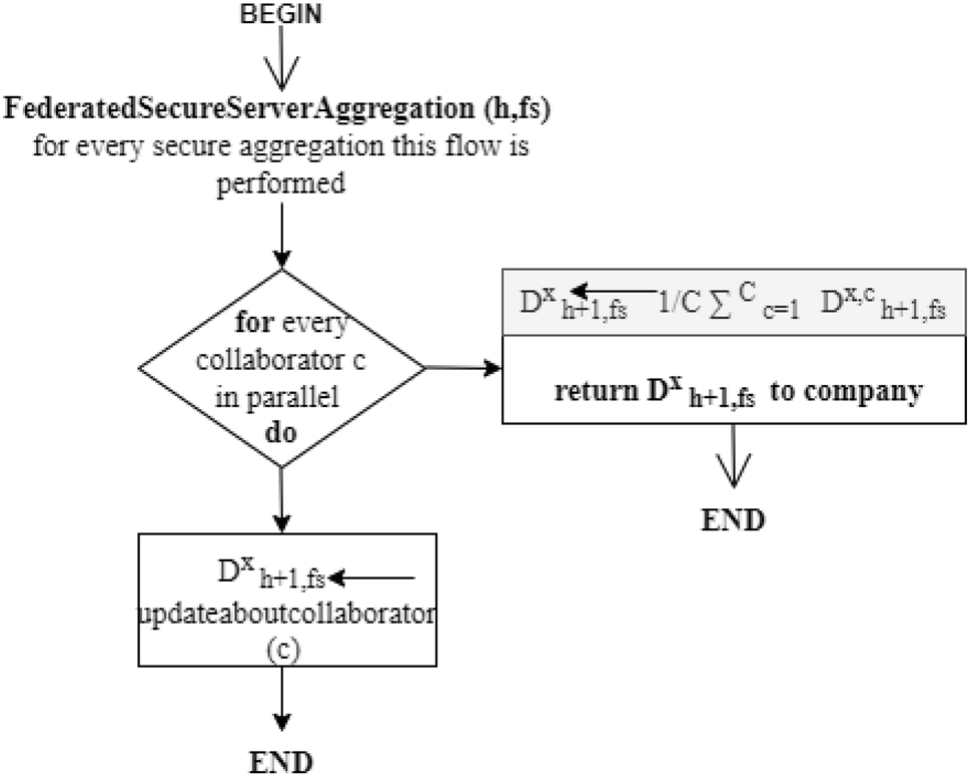
Flowchart for secure aggregation.

On every collaborator, the deployment of stochastic gradient descent (SGD) is used for optimization having the learning rate of *lr*. Every collaborator independently evaluates the average value on the local data for the present model *d*_*h*_ by utilizing the local batch for two or many more epochs ep, as shown in Eq. 3 (Figs. 4 and 5).

3 $$ m_{h}^{c} = \delta f_{c} (D_{h} ;v_{h} ) \, $$

**Figure 6:**
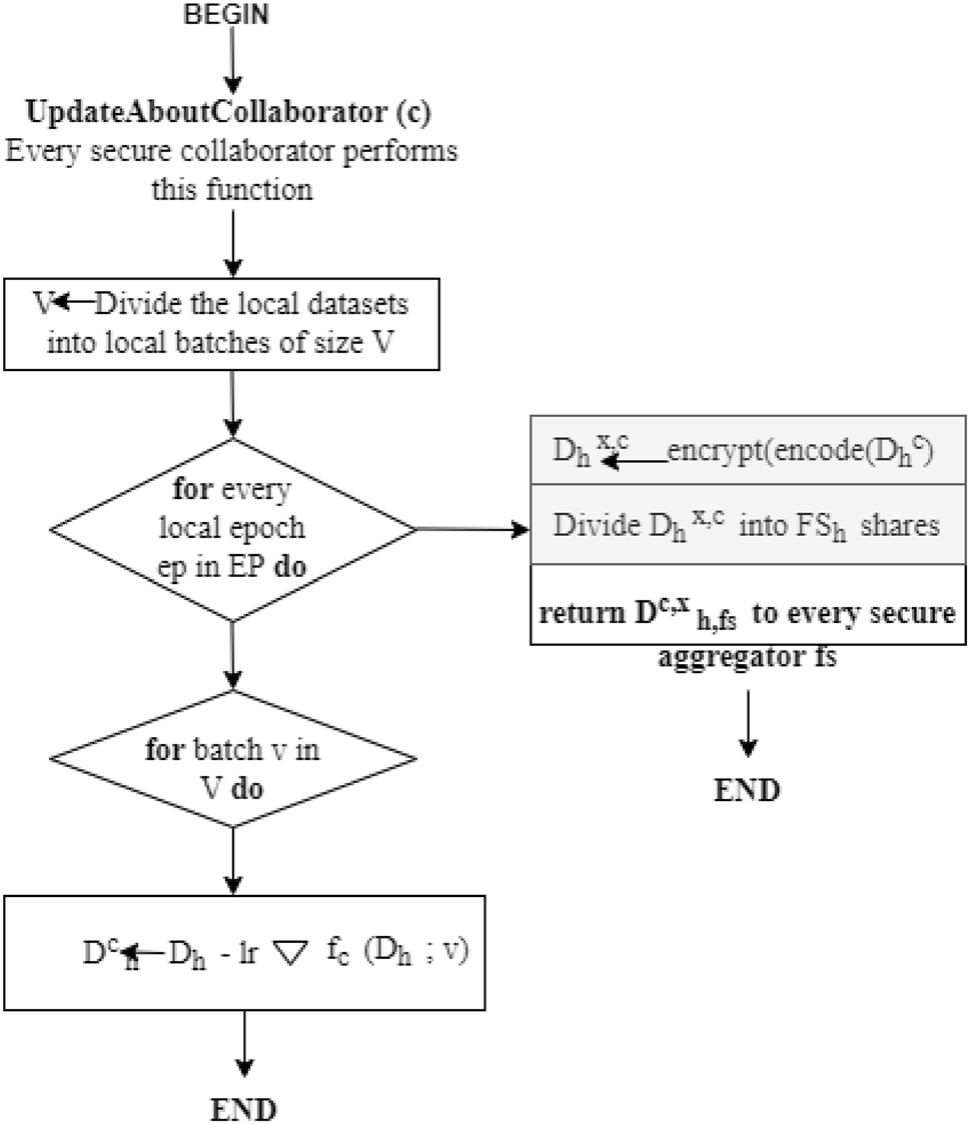
Flowchart for Collaborators at Cloud-Edge Terminal.

The training of the proposed work is shown as flowcharts in Figs. 4 and 6:

Furthermore, every collaborator took the move of localized gradient descent on the present model deploying the local data is shown in Eq. 4:

4 $$ \forall c;D_{h}^{c} \leftarrow D_{h} - lr\delta f_{c} (D_{h} ;v) $$

To safeguard the updates of the local model from any malicious event, the utilization of a security mechanism is used. This mechanism permits every collaborator c to divide its secret into multiple shares that are assigned to secure the global model aggregators so that every aggregator $fs = {1},...,FS_{h}$ has the value for encryption $d_{h,fs}^{x,c}$ and has nothing to learn regarding the secret. This phenomenon is called sharing the secret that is known well in cryptography, which facilitates the best efficiency with regard to an evaluation in contrast to existing cryptographic mechanisms.

The collection of finite fields is from $0,{1},...,P - {1}$ for every prime *P*, from which all the evaluations are taking place. Every collaborator c encodes the updates of local model *d*^*h*^ denoted as integers. Furthermore, every collaborator c divides the value encoded into shares of secret *FS*_*h*_. Every federated server aggregates fs, 1 <= *f s* <= *FS*_*h*_, which acquires any one of the secret shares that denote the value encrypted for the local model updates. Later on, every fs, combines the encrypted gradients. For every fs, the update of the final model becomes:

5 $$ \forall fs,D_{h + 1,fs}^{x} \leftarrow D_{h,fs}^{x} - lr\frac{1}{C}\sum\limits_{c = 1}^{C} {g_{h,fs}^{c} } $$

where $\frac{1}{C}\sum\limits_{c = 1}^{C} {g_{h,fs}^{c} }$. For every collaborator c, there is an update of the final model fo every secure server aggregator fs. The company finally regenerates the secret by adding updates to the finalized model from every aggregator fs. Later, the final updates of model parameters are decrypted and transmitted to the newly generated global model to every authorized collaborator. In this, the procedure will be repeated until and unless the maximum round is reached *h*_*maximum*_.

## Challenges and limitations of proposed framework

While utilizing federated learning and a distributed, secure collaborative framework for constructing an attack detection model in IoT domains offers numerous benefits, there are also several challenges and limitations to consider:

1. **Communication overhead:** Federated learning involves frequent communication between edge terminals and the central server for model updates. This can lead to increased communication overhead, especially in large-scale IoT deployments with a vast number of devices.
2. **Bandwidth constraints:** IoT devices often operate with limited bandwidth and intermittent connectivity. Transmitting model updates and aggregating data from diverse IoT domains may strain available bandwidth and exacerbate network congestion.
3. **Heterogeneity of IoT devices:** IoT ecosystems comprise a diverse range of devices with varying computational capabilities, communication protocols, and data formats. Ensuring interoperability and compatibility across heterogeneous devices poses challenges for federated learning implementations.
4. **Data imbalance and distribution:** In federated learning, data distribution across IoT domains may be uneven, leading to data imbalance and biased model training. Addressing data heterogeneity and ensuring representative sampling from diverse domains are crucial for achieving robust and generalizable attack detection models.
5. **Security and privacy risks:** While federated learning aims to preserve data privacy by keeping raw data on edge devices, there are inherent security and privacy risks associated with transmitting model updates and aggregating data on the central server. Mitigating these risks requires robust encryption, authentication, and access control mechanisms.
6. **Model synchronization and consistency:** Ensuring synchronization and consistency of model updates across distributed edge terminals is essential for maintaining the integrity of the global attack detection model. Handling delayed or lost updates, as well as addressing conflicts between divergent models, presents technical challenges in federated learning frameworks.
7. **Scalability and resource management:** As the number of IoT devices and domains increases, scalability becomes a concern for federated learning frameworks. Efficient resource management and allocation of computational resources are essential for scaling the framework to accommodate large-scale IoT deployments while maintaining performance and responsiveness.

Addressing these challenges requires careful consideration of technical, operational, and regulatory factors, as well as continuous innovation in federated learning techniques and distributed systems architecture. Despite these limitations, federated learning remains a promising approach for building effective attack detection models in IoT domains while preserving data privacy and security.

## Results and discussions

In this section, the implementation and computations of the proposed work are presented. Firstly, the experimental environment is discussed to depict the results. The performance evaluation of the proposed work is presented.

### BoT-IoT dataset

In this subsection, the use of the most popular cyber security dataset known as BoT-IoT is available for the research. It comprises normal traffic and malicious attack scenarios that include DoS, DDoS, reconnaissance, and data theft. The numerous testbeds are generated in which normal traffic on the network is composed of weather stations, smart refrigerators, and smart lights. Various researchers^23^ have developed a method for capturing the packets on the network and extracting features. In this dataset, packets in the network are captured by deploying the Tshark (https://www.wireshark.org/docs/man-pages/tshark.html.) tool, whereas the features of the network were extracted by deploying the tool named Argus (https://openargus.org/.). Also, the new features were obtained based on the number of transaction flows of connections in the network in connection to the sliding window of 200. The other authors in^24^ have also confirmed the method of extracting the features for malicious classification of the traffic on the network. There are in all 43 features were extracted from the packets of the network to discuss the sample’s network traffic behavior. The BoT-IoT has samples of 477 normal traffic and 3668045 malicious attacks.

In this paper, seven duplicate features are removed from the dataset: a. pkSeqID, b. flgs, c. *f lgs*_*n*_*umber*, d. proto, e. state, f. min, g. max. As pkSeqID is defined as the identification of the sequence numbers allocated to the packets in the network; flgs, *f lg*_*n*_*umber* are the flow state flags that are observed in the transactions and representation of features in flags done numerically respectively; state and proto are the transaction protocols in data transmission that are represented textually. In this case, a total of 36 features are used to depict the samples of traffic on the network. The values of these features were scaled to numbers between 1 and 0 for the efficient training of the neural network. This can be performed by using the min-max normalization shown as:

6 $$ a_{norm} = \frac{{a - a_{min} }}{{a_{max} - a_{min} }} $$

where a is the feature vector for traffic on the network; *a*_*max*_ and *a*_*min*_ are depicted as the maximum and minimum values of a.

The table below shows the cyberattacks describing the major categories of the BoT-IoT dataset:

1. **Probing attacks:** Attacks known as probing are hostile actions that examine remote computers, or so-called "fingerprint- ing," to gain more information about users. These attacks are divided into various subcategories, the first one relies on the actions performed during the probing and the second one relies on the target of gathered information.
2. **Denial of Service (DoS):** DoS refers to malicious actions that aim to interrupt service and prevent legitimate traffic from using it. The dataset’s DDoS and DoS attack types are outlined as follows: A collection of infected machines known as Bots conduct Distributed Denial of Service (DDoS) and DoS attacks against a remote computer, typically a server. Such attacks are conducted with the intention of interfering with services that authorized users can access.
3. **Information Theft:** Information theft refers to a class of attacks where a perpetrator tries to undermine a machine’s safety in order to obtain confidential information. The dataset’s information stealing attack categories are characterized as follows: Depending on the goal of the attack, information theft attacks can be divided into different subcategories. The first subdivision is information theft. In data theft attacks, a remote machine is targeted and attempted to be compromised in order to gain access to information that can then be downloaded to the remote attacking system. Keylogging is indeed the second category. An attacker breaches a remote host during keylogging operations to capture a user’s keystrokes and perhaps steal sensitive credentials.

### Performance evaluation

The implementation of the proposed framework is utilized by using a library to secure the federated data called Pysyft, which is developed on top of PyTorch. The proposed framework is deployed using TensorFlow Federated (TFF), a Python 3 open-source framework used by federated learning. At first, the data is coded, and afterward, the proposed framework is compiled into virtual machine byte code. Once the compilation is done, the byte code is obtained. The experiments are run on the Jupyter Notebook, which is deployed using the *NVIDIADGX*^*TM*^ package repositories.

#### Preprocessing of BoT-IoT Dataset

The framework for zero-touch botnet attacks in federated learning for five edge-based IoT devices using the BoT-IoT dataset. In this, the devices in IoT have very few resources computed and very limited utilization of memory space used for storing the data. Thus, the traffic on private networks is generated by the IoT inside the same network that is stored in edge-based IoT devices for easy processing. The five IoT-based edge devices include IoT-Domain1, IoT-Domain2, IoT-Domain3, IoT-Domain4, and IoT-Domain5.

**Figure 7:**
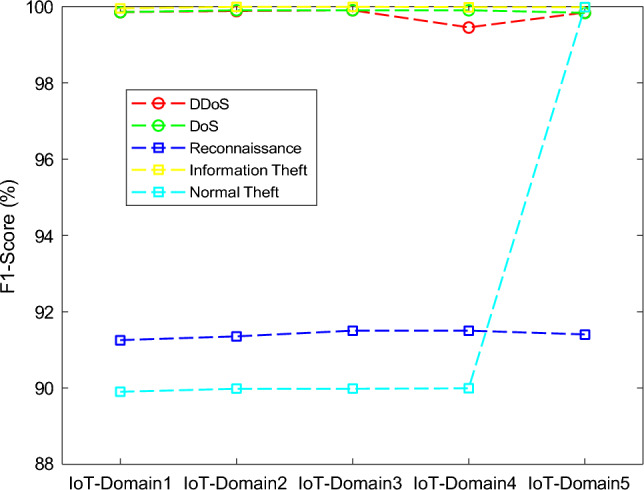
F1 implementation with five different IoT domains.

**Figure 8:**
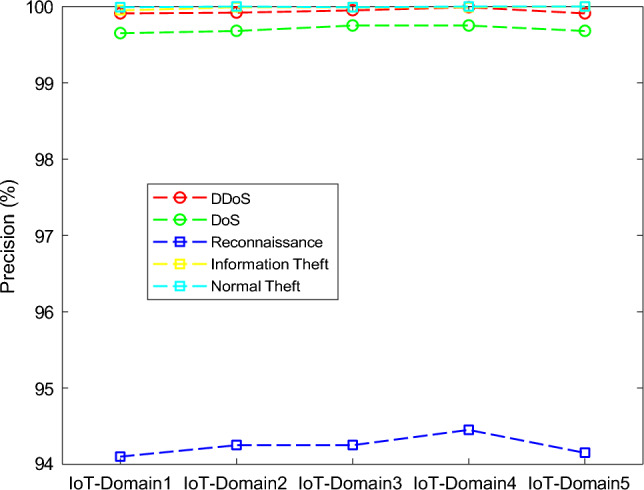
Precision implementation with five different IoT domains.

**Figure 9:**
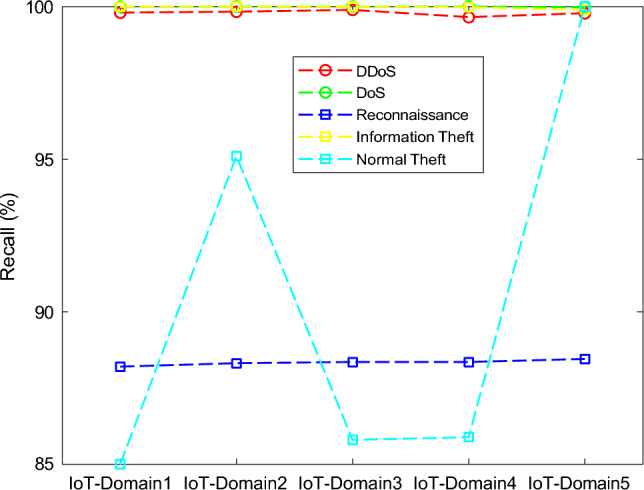
Recall implementation with five different IoT domains.

**Figure 10:**
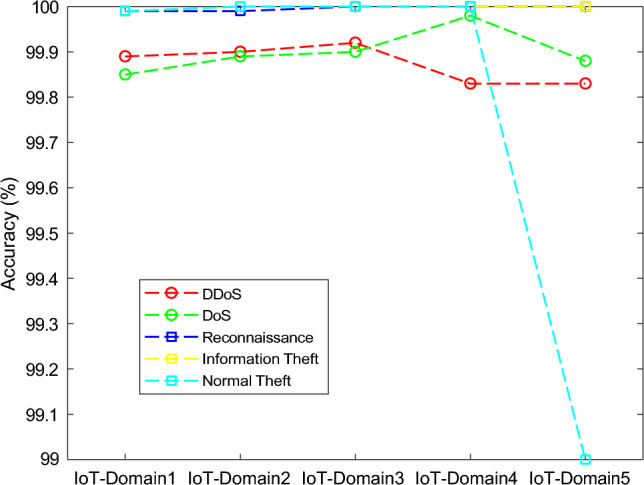
Accuracy implementation with five different IoT domains.

The results of the proposed federated learning model are represented in Figs. 7, 8, 9 and 10. The results show that the proposed model acquires the maximum performance in detecting the normal traffic on the network and various attacks in the five IoT domains. Thus, the updates of the local model from the devices in IoT extending the epochs do not result in achieving maximum performance.

Using the BoT-IoT dataset, Tables 1 and 2 display the splitting of testing and training data amongst the various IoT domain devices. Without concern for data privacy, the category of traffic on the network was missing in every IoT domain device, which depicted the capability of a federated learning model for the detection of next-generation malware. Furthermore, there is no sample of DDoS, DoS, Reconnaissance, Information theft, or traffic on the network included in all the domains. To describe the real-life experiments, imbalanced the splitting of training and splitting the unidentified data across the different categories and five domains. The performance evaluation of the federated learning model was computed using the different sets of testing data in every IoT-domain device.

**Table 1:** Training Data Splitting in BoT-IoT Dataset.

Category	IoT-Domain1	IoT-Domain2	IoT-Domain3	IoT-Domain4	IoT-Domain5
DDoS	0	337,162	337,163	337,163	337,163
DoS	288,752	288,752	288,752	0	288,752
Normal	84	84	84	84	0
Reconnaissance	15,979	0	15,979	15,979	15,979
Information Theft	13	14	0	14	14

**Table 2:** Testing Data Splitting in BoT-IoT Dataset.

Category	IoT-Domain1	IoT-Domain2	IoT-Domain3	IoT-Domain4	IoT-Domain5
DDoS	115,413	115,382	115,459	115,570	115,406
DoS	99,090	99,303	99,193	99,016	99,354
Normal	31	26	34	32	26
Reconnaissance	5572	5398	5420	5490	5318
Information Theft	5	2	5	3	7

### Experimentation

The model named Deep Neural Networks was trained and tested with the dataset chosen to decide the optimized framework of the neural network for the effective classification of traffic on the network. Centralized learning, splitting learning, and federated learning models were proposed for the autonomous detection of attacks in various domains. The use of TensorFlow and Pandas framework for the Deep Neural Network model was proposed, and the IBM framework was used for federated learning in the federated learning model. The training of the model was utilized using the Visual Studio IDE that is running on Ubuntu 16.04LTS with the said specifications with RAM 16GB, processor intel Core i7 8th Generation Quad-Core Processor with the 64-bit operating system. The utilization of a federated learning model in IoT-based domain devices was evaluated by deploying different terminals. Lastly, the performance classification was computed based on the metrics: accuracy, precision, recall, and F1-score.

The framework of the model is composed of an input layer, a hidden layer, and an output layer. All the layers are comprised of various neurons. In the training data, the number of neurons is similar to the features of traffic on the network. A total of 36 features are extracted in the BoT-IoT dataset. When the DNN model was trained, the total number of input layer neurons was 36. During the experimentation, the total number of hidden layers and neurons is decided with the help of experiments. The number of hidden layers is diverged between 2 and 5, whereas the number of neurons hidden is diverged between 30 and 120 at the interval of 30. The total five categories of attack and normal are there in the BoT-IoT dataset. Thus, the deep neural network is trained with a batch size of 256 and 50 epochs to minimize the time spent during the training process and avoid the model being overfitted.

The performance classified based on the different models was tested and computed with the testing data in the IoT-domains devices on the basis of accuracy, precision, recall, and F1-score and the comparison is done with the baseline models centralized deep learning model^25^, Localized deep learning model^26^ and federated deep learning model.

Where True Positive (TP) is defined as the total number of samples in network traffic that are correctly classified as positive in the positive class; False Positive (FP) is defined as the total samples in network traffic that are not classified as positive in the negative class; True Negative (TN) is defined as the samples in network traffic that are classified as negative in the negative class; False Negative (FN) is defined as the total samples in network traffic that are not classified as positive in the negative class.

In Table 3, the effectiveness of the federated learning model in the five IoT-domains devices. The performance of all the domains is trained and tested with the BoT-IoT dataset. The features of traffic on the network of the devices in the IoT-based domain in the edge.

**Table 3:** Based on Bot-IoT dataset performance classification of federated learning model.

Domains	Metrics (%)	DDoS	DoS	Reconnaissance	Information Theft	Normal Traffic
IoT-Domain1	Accuracy	99.89	99.85	99.99	99.99	99.99
	Precision	99.91	99.65	94.10	99.95	99.99
	Recall	99.80	99.99	88.20	99.99	85.00
	F1-Score	99.85	99.85	91.25	99.95	89.90
IoT-Domain2	Accuracy	99.90	99.89	99.99	100.00	100.00
	Precision	99.92	99.68	94.25	99.99	100.00
	Recall	99.83	100	88.31	100.00	95.10
	F1-Score	99.88	99.90	91.35	99.99	89.98
IoT-Domain3	Accuracy	99.92	99.90	100.00	100.00	100.00
	Precision	99.95	99.75	94.25	99.99	99.99
	Recall	99.89	100.00	88.35	100.00	85.80
	F1-Score	99.90	99.90	91.50	99.99	89.98
IoT-Domain4	Accuracy	99.83	99.98	100.00	100.00	100.00
	Precision	99.99	99.75	94.45	99.99	100.00
	Recall	99.65	100.00	88.35	99.98	85.89
	F1-Score	99.45	99.90	91.50	99.98	89.99
IoT-Domain5	Accuracy	99.83	99.88	100.00	100.00	99.00
	Precision	99.91	99.68	94.15	100.00	100.00
	Recall	99.78	99.98	88.45	99.90	100.00
	F1-Score	99.84	99.83	91.40	99.98	99.98

Based on the accuracy, precision, recall, and f1-score, the performance classification of centralized, localized, and proposed deep learning models is computed with the testing data in the five different domains. The metrics used for the performance evaluation in our study are:

1. ***Accuracy:*** This statistic aids in figuring out a classifier’s accuracy. It establishes how many accurate predictions the model built. It is the proportion between the number of accurate predictions and all of the model’s other predictions.
2. ***Precision:*** It is the percentage of correctly predicted values to all correctly predicted values as determined by the classification model.
3. ***Recall:*** It describes the overall number of records for a given class that can be correctly predicted using whatever data is available.
4. ***F1-Score:*** also referred to as the mean of recall and precision, employs recall and accuracy to evaluate the model thoroughly.

**Figure 11:**
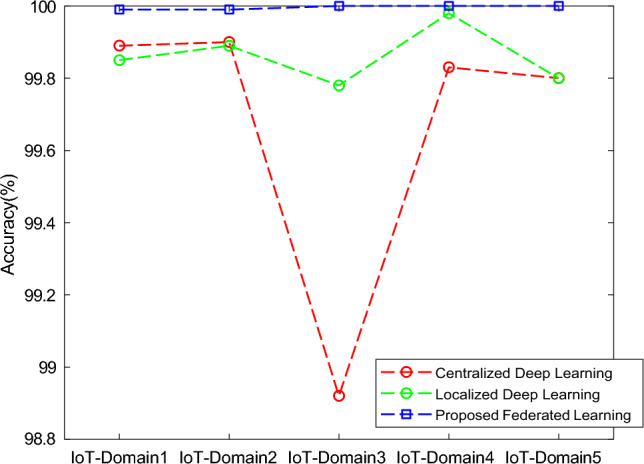
Comparative analysis of Accuracy with baseline models for different five IoT domains.

In Fig 11, the analysis of centralized and localized deep learning models is compared with the proposed federated learning model. In this, the accuracy of the proposed model is increased as the total number of iterations or rounds is extended up to 30 when the training is done with the dataset. At the end of 30 iterations, the proposed model had an accuracy of 100.00%.

**Figure 12:**
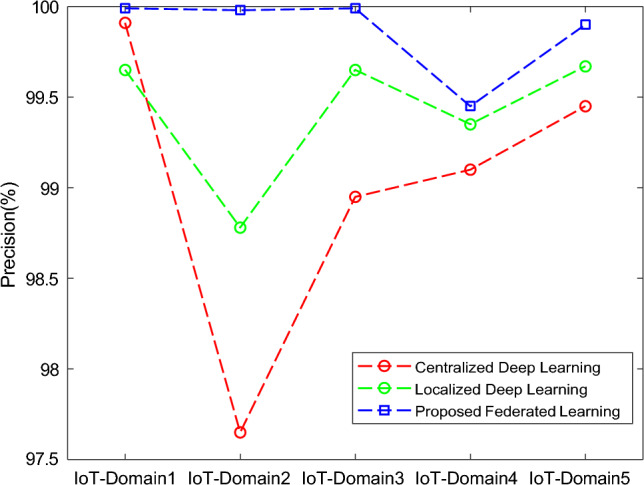
Comparative analysis of Precision with baseline models for different five IoT domains.

In Fig 12, the analysis of centralized and localized deep learning models is compared with the proposed federated learning model. In this way, the precision of the proposed model is increased as the total number of iterations or rounds is extended up to 30 when the training is done with the dataset. At the end of 30 iterations, the proposed model had an accuracy of 99.99%.

**Figure 13:**
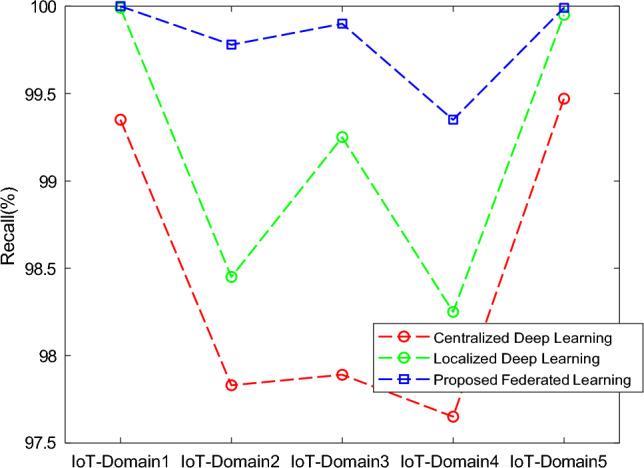
Comparative analysis of Recall with baseline models for different five IoT domains.

In Fig 13, the analysis of centralized and localized deep learning models is compared with the proposed federated learning model. In this, the recall of the proposed model is increased as the total number of iterations or rounds is extended up to 30 when the training is done with the dataset. At the end of 30 iterations, the proposed model had an accuracy of 99.90%.

**Figure 14:**
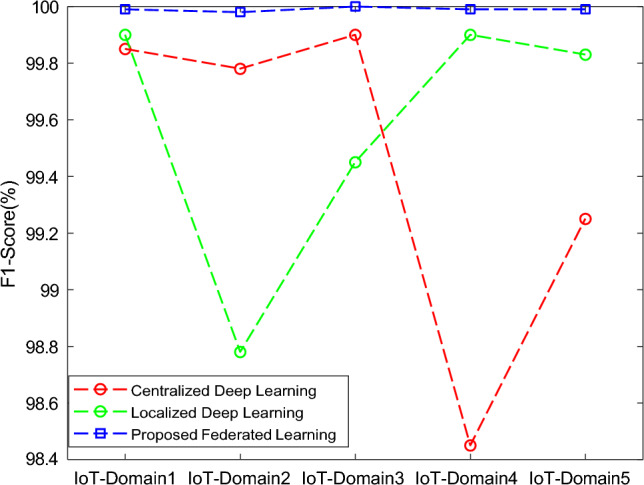
Comparative analysis of F1-Score with baseline models for different five IoT-domains.

In Fig 14, the analysis of centralized and localized deep learning models is compared with the proposed federated learning model. In this way, the f1-score of the proposed model is increased as the total number of iterations or rounds is extended up to 30 when the training is done with the dataset. At the end of 30 iterations, the proposed model had an accuracy of 99.99%.

In Table 4, the comparison of the proposed model is made with the baseline models, and we have observed that the performance analysis of the proposed framework is relatively higher than the baseline models. The other models have significantly lower rates of detection of attacks and maximum false alarm rates. Because all of them are trained with insufficient network traffic and very few attack scenarios in IoT-based devices, the classification performance of the localized deep learning model is lower. The centralized and localized deep learning models degrade the performance due to the updates of the local model to the server being less than one round of iteration. That is why both of these models are not compatible to be implemented for attack detection in the proposed framework. The proposed framework can detect the attack with the highest classified performance while having the least communication cost and less memory to store the data. Therefore, it acquires maximum performance. The time required to train the proposed model is due to the complexity of the proposed method.

**Table 4:** Comparison of federated learning model with the baseline models.

Domains	Metrics (%)	Centralized Deep Learning Model	Localized Deep Learning Model	Federated Learning Model
IoT-Domain1	Accuracy	99.89	99.85	99.99
	Precision	99.91	99.65	94.80
	Recall	99.35	99.99	100.00
	F1-Score	99.85	99.90	99.99
IoT-Domain2	Accuracy	99.90	99.89	99.99
	Precision	97.65	98.78	99.98
	Recall	97.83	98.45	99.78
	F1-Score	99.78	98.78	99.98
IoT-Domain3	Accuracy	98.92	99.78	100.00
	Precision	98.95	99.65	99.99
	Recall	97.89	90.00	99.90
	F1-Score	99.90	99.45	100.00
IoT-Domain4	Accuracy	99.83	99.98	100.00
	Precision	99.10	99.35	99.45
	Recall	97.65	98.25	99.35
	F1-Score	98.45	99.90	99.99
IoT-Domain5	Accuracy	99.80	99.80	100.00
	Precision	99.45	99.67	99.90
	Recall	99.47	99.95	99.99
	F1-Score	99.25	99.83	99.99

## Conclusion

In this paper, a privacy-preserving attack detection model is introduced within the context of federated learning, specifically tailored for zero-touch networks across five distinct IoT domains. To validate the effectiveness of our proposed model, we leverage the BoT-IoT dataset, aiming to showcase its maximum performance. In the evaluation, our model is systematically compared against baseline models, namely centralized and localized deep learning approaches. The centralized model, characterized by the aggregation of data, demonstrated superior performance. However, a significant drawback was its inability to secure the traffic (data) flowing into different domains, resulting in increased delays and extended training times. On the other hand, the localized model addressed the security concern associated with the centralized approach but suffered from diminished performance when compared to the centralized model. Our proposed model strategically overcomes the limitations of both centralized and localized models, achieving not only maximum performance but also minimizing delays and enhancing accuracy, precision, recall, and F1-score. Consequently, our model emerges as an effective solution for attack detection across diverse IoT domains. Looking ahead, we envision further optimization of performance by incorporating advanced algorithms tailored for the detection of attacks in the evolving landscape of 6G networks. This continuous refinement will ensure our model remains at the forefront of safeguarding privacy and enhancing security in future network environments.

## Data Availability

All data generated or analyzed during this study are included in this published article.
